# Assessment of the World Health Organization’s HIV Drug Resistance Early Warning Indicators in Main and Decentralized Outreach Antiretroviral Therapy Sites in Namibia

**DOI:** 10.1371/journal.pone.0166649

**Published:** 2016-12-01

**Authors:** Nicholus Mutenda, Alexandra Bukowski, Anne-Marie Nitschke, Tuli Nakanyala, Ndapewa Hamunime, Tadesse Mekonen, Francina Tjituka, Greatjoy Mazibuko, Samson Mwinga, David Mabirizi, Evans Sagwa, Rosalia Indongo, Natalie Dean, Michael R. Jordan, Steven Y. Hong

**Affiliations:** 1 Directorate of Special Programmes, Republic of Namibia Ministry of Health and Social Services, Windhoek, Namibia; 2 Department of Public Health and Community Medicine, Tufts University School of Medicine, Boston, Massachusetts, United States of America; 3 Systems for Improved Access to Pharmaceuticals and Services (SIAPS), Management Sciences for Health, Windhoek, Namibia; 4 United States Agency for International Development, Windhoek, Namibia; 5 Department of Biostatistics, University of Florida, Gainesville, Florida, United States of America; 6 Division of Geographic Medicine and Infectious Diseases, Tufts Medical Center, Boston, Massachusetts, United States of America; National and Kapodistrian University of Athens, GREECE

## Abstract

**Background:**

The World Health Organization (WHO) early warning indicators (EWIs) of HIV drug resistance (HIVDR) assess factors at individual ART sites that are known to create situations favourable to the emergence of HIVDR.

**Methods:**

In 2014, the Namibia HIV care and treatment program abstracted the following adult and pediatric EWIs from all public ART sites (50 main sites and 143 outreach sites): *On-time pill pick-up*, *Retention in care*, *Pharmacy stock-outs*, *Dispensing practices*, and *Viral load suppression*. Comparisons were made between main and outreach sites and between 2014 and 2012 using the Wilcoxon signed-rank test in a matched analysis.

**Results:**

The national estimates were: *On-time pill pick-up* 81.9% (95% CI 81.1–82.8) for adults and 82.4% (81.3–83.4) for pediatrics, *Retention in care* 79% retained on ART after 12 months for adults and 82% for pediatrics, *Pharmacy stock-outs* 94% of months without a stock-out for adults and 88% for pediatrics, and *Dispensing practices* 0.01% (0.001–0.056) dispensed mono- or dual-therapy for adults and 0.01% (0.001–0.069) for pediatrics. *Viral load suppression* was significantly affected by low rates of *Viral load completion*. Main sites had higher *On-time pill pick-up* than outreach sites for adults (p<0.001) and pediatrics (p<0.001), and no difference between main and outreach sites for *Retention in care* for adults (p = 0.761) or pediatrics (p = 0.214). From 2012 to 2014 in adult sites, *On-time pill pick-up* (p = 0.001), *Retention in care* (p<0.001), and *Pharmacy stock-outs* (p = 0.002) worsened. In pediatric sites, *On-time pill pick-up* (p<0.001) and *Pharmacy stock-outs* (p = 0.012) worsened.

**Conclusions:**

Results of EWIs monitoring in Namibia provide evidence about ART programmatic functioning and contextualize results from national surveys of HIVDR. These results are worrisome as they show a decline in program performance over time. The national ART program is taking steps to minimize the emergence of HIVDR by strengthening adherence and retention of patients on ART, reducing stock-outs, and strengthening ART data quality.

## Introduction

### Background

As of 2015, 15 million people were receiving antiretroviral therapy (ART) for treatment of HIV infection globally. [1] This rapid scale up of ART is the result of national and international public health efforts. However, the widespread use of ART worldwide poses a great risk for the emergence of population-level HIV drug resistance (HIVDR) without a proper focus on quality of ART delivery and surveillance of HIVDR.

The Joint United Nations Programme on HIV/AIDS (UNAIDS) released new global HIV response targets in 2014 that called for further impact on the HIV epidemic by treating as early as possible and monitoring progress through harmonized targets. [2] These are referred to as the “90-90-90 targets” to end the HIV pandemic by 2030: for 90% of people living with HIV (PLHIV) to know their HIV status, 90% of PLHIV who know their status to receive life-saving ART, and 90% of PLHIV on ART to have suppressed viral load by 2020.

With its "treat-all" recommendation in 2015, the World Health Organization (WHO) recommended that all infected individuals with HIV should begin ART as soon after diagnosis as possible. [3] This recommendation will undoubtedly lead to a decrease in HIV incidence. However, paradoxically, we may see an increase in HIV drug resistance (HIVDR) amongst those infected. [4–5] HIVDR is important as it can impact achieving the third “90”. Therefore, efforts to measure and respond to HIVDR are critical to achieve sustained population-level viral suppression and ultimately attain the UNAIDS goal of HIV elimination by 2030.

To address the risk of HIVDR, the WHO developed a global HIVDR surveillance and monitoring strategy based on public health principles, which was updated in 2015 to include 4 key elements: 1.) *Monitoring of Early Warning Indicators (EWI) of HIVDR*, 2.) *Surveillance of HIVDR in populations initiating ART*, 3.) *Surveillance of acquired HIVDR in populations on ART*, 4*s*, and 4.) *Surveillance of HIVDR in children <18 months of age*. [6] EWIs of HIVDR have been successfully integrated into the national HIV program monitoring and evaluation in Namibia. The methods and findings of the assessment of Namibia’s 2014 EWIs are described herein. This 2014 EWI monitoring round represents the most robust and comprehensive evaluation performed in Namibia to date.

### HIVDR Early Warning Indicators

HIVDR EWIs assess the functioning of national HIV programmes by monitoring factors at individual ART sites that are known to create situations favourable to the emergence of HIVDR. In many limited resource settings, HIVDR testing is not routinely available nor recommended for guiding first- and second-line ART selection. Thus, EWIs use routinely-collected clinical and pharmacy data to alert ART sites and programs to situations which may favor HIVDR when ART sites are not achieving globally-recommended standard targets. Using these data, national programmes may tailor interventions to optimize standard HIV care and therefore reduce the risk of emergence of population-level HIVDR. HIVDR may not necessarily result immediately if an indicator shows non-optimal performance; however, achieving the best possible performance as measured by these indicators will help to minimize preventable HIVDR and therefore maximize long-term population-level viral suppression. [6] WHO updated its EWI guidance in 2012 [7], and as of 2016, 55 countries worldwide had reported clinic-level data to the WHO. [8]

### HIV in Namibia

Namibia is a low-middle income country in sub-Saharan Africa, where approximately 210,000 people are living with HIV in a population of 2.4 million [9–10]. Among pregnant women aged 15–49 years of age, approximately 16.9% are infected with HIV-1. [11] However, there is great regional variability in Namibia, with an estimated prevalence of up to 36% in the most-affected areas. The epidemic is predominantly spread through heterosexual contact. [11]

### ART rollout

In Namibia, ART has been available to patients in the private sector since 1997 and in the public sector since 2003, where it is available free-of-charge. Namibia’s ART coverage for adult and pediatric patients is 58%, with 143,805 eligible patients on ART as of December 2015. ART is currently available at 258 public ART sites throughout the country (221 at the time of this EWI survey). [unpublished data from Ministry of Health and Social Services] Of these sites, the national ART program considers 50 to be “main” ART sites, which dispense ART independently. The remainins sites consist of satellite/“outreach” sites and Integrated Management of Adolescent and Adult Illness (IMAI) sites.

All public ART sites in Namibia have access to both first- and second-line regimens, which are available using a population-based model of care according to WHO guidance used during the time of these EWIs. [12] The decision to initiate ART is based on WHO clinical staging and/or CD4 cell count ≤ 500 cells/mm^3^. The following subpopulations are also eligible for ART in Namibia: all HIV-infected pregnant women, children and adolescents living with HIV and aged less than 15 years, HIV/Hepatitis B co-infected individuals, HIV-positive sero-concordant couple aspiring to have a baby, and the HIV-infected partner in a sero-discordant relationship. At all public ART sites, routine viral load monitoring is recommended six and 12 months after ART initiation, and every 12 months thereafter (every 6 months for children/adolescents <18 years). [13] Two national databases are utilized by the HIV program in the public sector in Namibia: 1.) the Electronic Dispensing Tool (EDT), a standardized pharmacy record system, and 2.) the electronic Patient Management System (ePMS), a clinical record system.

The private sector provides care for approximately 18% of patients on ART in Namibia, and its model of care differs from that of the public sector. [14]

### Early Warning Indicators in Namibia

EWIs were introduced in Namibia in 2009, when five EWIs were piloted at nine ART sites. [15] In 2010, Namibia analyzed these same EWIs, scaling up from nine to 33 ART sites. [16] In 2012, Namibia utilized the updated WHO guidelines for EWI data abstraction at 50 ART sites. [17]

The annual EWI monitoring led to public health actions targeted at optimizing quality of care, improving of ART record systems, engagement of ART sites, and initiation of operational researches to improve adherence and retention. Lessons learned from the 2012 EWI abstraction exercise were used to inform training and optimize data abstraction for this 2014 EWI exercise.

## Methods

### Early Warning Indicator definitions and targets

The Namibia HIVDR technical working group (TWG), through a consultative process, abstracted the following EWIs in 2014 according to WHO definitions (Table 1): *On-time pill pick-up*, *Retention in care*, *Pharmacy stock-outs*, *Dispensing practices*, *Viral load suppression at 6 months*, and *Viral load completion at 6 months*. Namibia used a country-specific definition for viral load suppression, due to routine viral load testing six months after ART initiation throughout the country.

**Table 1:** Selected 2014 WHO Early Warning Indicator Definitions (Numerator/Denominator) and Targets.

Early Warning Indicator	Definitions (Numerator/Denominator)	Targets^#^
**1. On-time pill pick-up**	Numerator: number of patients picking up their ART on time* at first drug pick-up after a defined baseline pick-up date.	Red: <80% of patients picking up pills on-time
	Denominator: number of patients who picked up drugs on or after the designated EWI sample start date.§	Amber: 80–90% of patients picking up pills on-time
		Green: >90% of patients picking up pills on-time
**2. Retention in care**	Numerator: number of adults or children who are still alive and on ART 12 months after initiating treatment.	Red: <75% retained after 12 months of ART
		Amber: 75–85% retained after 12 months of ART
	Denominator: total number of adults or children who initiated ART who were expected to achieve 12-month outcomes within the reporting period, including those who have died since starting therapy, those who have stopped therapy, and those recorded as lost to follow-up^†^ at month 12.^∞^	Green: >85% retained after 12 months of ART
**3. Pharmacy stock-outs**	Numerator: number of months in the designated year in which there were no stock-out^‡^ days of any (adult or pediatric) ARV drug routinely used at the site.	Red: <100% of a 12-month period with no stock-outs
	Denominator: 12 months.	Green: 100% of a 12-month period with no stock-outs
**4. Dispensing practices**	Numerator: number of patients (adults or children) who pick up form the pharmacy, a regimen consisting of one or two ARVs.	Red: >0% dispensing of mono- or dual therapy
	Denominator: number of patients (adults or children) picking up ART on or after the designated EWI sample start date. Sampling continues until the full sample size is reached.	Green: 0% dispensing of mono- or dual therapy
**5. Viral load suppression at 6 months**^	Numerator: number of patients receiving ART and a viral load at the site after the first 6 months of ART whose viral load is <1000 copies/mL.	Red: <75% of viral loads suppressed
		Amber: 75–90% of viral loads suppressed
	Denominator: total number of ART starters from 1 October 2012 who have 6-month viral load results available (first viral load completed between 3–12 months from ART initiation, and lab date on or after 1 October 2013).	Green: >90% of viral loads suppressed
**5a. Viral load completion at 6 months**^	Numerator: number of patients retained on ART at the site after the first 6 months of ART who had viral load results available	Red: <70% of eligible patients completing 6-month viral load results^+^
	Denominator: total number of patients at the site retained on ART 6 months after ART initiation	Green: ≥70% of eligible patients completing 6-month viral loads

ART—Antiretroviral therapy

ARV—Antiretrovirals

* On-time pill pick-up: Pick up pills no more than two days late on their first pick-up after a baseline pick-up

^§^ EWI sample start date: The date designated as the start of the sampling. The sample start date is fixed by the HIVDR Working Group.

^†^ Lost to follow-up: Patients who had not returned to the pharmacy or clinic ≤90 days after the last ART run-out date during the 12-months after the date of ART initiation were classified as LTFU. Stopping therapy without restarting was classified as not LTFU if the patient continued to attend clinic appointments.

^∞^ Transfers of care to another site were excluded from the denominator.

^‡^ Stock-out: Any occurrence of zero stock of a routinely-used ARV drug at the site at which the patient routinely picks up ARVs.

^#^ Adult and pediatric targets are the same.

^ Due to routine data collection of viral load at 6 months, Namibia chose to monitor *Virological suppression at 6 months* instead of the WHO recommendation of 12 months. The same 12-month targets were used for the analysis.

^+^ Target adapted from WHO Strategic Information Guideline [15]

Table adapted from WHO HIV Drug Resistance EWI guidance report [4]

The performance of ART sites was rated for each EWI according to WHO recommended targets (Table 1). [6–7] As WHO targets were not available for *Viral load suppression at 6 months*, Namibia used WHO’s 12-month targets as international literature reflected similar viral load suppression rates at 6 and 12 months. [18] The targets utilize three performance classifications: red (poor performance, below desired level), amber (fair performance, not yet at desired level), and green (excellent performance, achieving desired level). The targets also allow for a “grey” classification if a site does not have data available to monitor a specific EWI. For *Viral load suppression at 6 months*, if *Viral load completion at 6 months* for that site was <70% (red), the site received grey for *Viral load suppression at 6 months*, meaning that the record keeping system did not support valid results. EWIs were monitored separately for adult and pediatric patients. [19]

### Ethics statement

Ethical review was not required as this data were public health surveillance data abstracted from routinely collected Ministry of Health and Social Services (MoHSS) medical records. Only anonymized data were abstracted from the medical records for public health surveillance purposes. After discussion with the Tufts Medical Center institutional review board and the MoHSS, it was determined that because this was routine public health de-identified data analyzed within the MoHSS in Namibia, no formal written waiver was necessary.

### Site selection and data abstraction

WHO recommends that EWIs be collected routinely from all ART sites within a country, or from a large number of representatively sampled sites, in order to characterize program-level performance. In Namibia, all 50 main public ART delivery sites and their respective outreach sites (>95% of all patients receiving ART from public sites) were included in the EWI abstraction in 2014, which includes those that participated in EWI abstraction in 2012. Individual outreach site data were disaggregated from main site data and were monitored both individually and as part of their main sites. EWI data abstraction was conducted centrally in June 2014 by a trained data abstraction team formed by the TWG in collaboration with both the WHO and the Namibia MoHSS.

Both national ART patient databases in Namibia were used: the Electronic Dispensing Tool (EDT) and the electronic Patient Management System (ePMS). Data for *On-time pill pick-up*, *Retention in care*, and *Dispensing Practices* were abstracted from EDT through automatic queries into MS Excel. Data for *Pharmacy stock-outs* were calculated from monthly site-level ART reporting. Data for *Viral load suppression at 6 months* were abstracted centrally from ePMS into MS Excel.

### Data quality assessment

Similar to past EWI monitoring rounds in Namibia [15–17], data quality assessments were implemented throughout the EWI process on the data queried centrally from the two national databases. Three elements of data quality were considered in the assessments: data reliability, data completeness, and data consistency. Data reliability, which is an assessment of the quality of the abstraction, was assessed by confirming at least 10% of the centrally-queried data to the existing data in the EDT. Data completeness was assessed from the centrally-queried data and varied across sites for specific variables; sites that had no available data for a specific EWI were given a “grey” classification for that variable. Lastly, assessment of data consistency, which determines the optimal source of data for each variable, had been previously performed during the first pilot of EWIs in Namibia. [15] These previously determined standards continue to be used for data abstraction: EDT data are considered the gold standard for pharmacy data, while ePMS and paper records (Patient Care Booklets) are considered the gold standard for patient clinical information.

### Data validation

Data queried from each of the two national databases were validated against information in the other to remedy various data inconsistencies, such as ambiguous patient statuses, file duplicates, and incomplete fields due to erroneous data entry.

Three data validation steps were performed for the first time in 2014 to further improve data quality: 1) validation of all missing patient unique numbers, 2) classification of all patients with a “lost” status (missing from clinic between 30–90 days), and 3) removal of duplicate patient entries.

### Sample size

To achieve a representative site-level result for each EWI, WHO recommends data abstraction on a minimum number of consecutive patients to provide a 95% confidence interval (CI) of ±7%. The target sample size for each EWI is based on the number of eligible patients according to WHO guidance, and the Namibian methods discussed herein have been previously detailed by Jonas et al. [16–17] For *On-time pill pick-up* and *Dispensing practices*, the number of eligible patients at each site to be sampled was the number of patients who were “active” on ART at the time of the sample start date (1 January, 2013). All sites began abstraction from the sample start date and abstracted data until the appropriate sample size for each site was reached, and data were oversampled by 20% to account for potential censoring of patients. For *Retention in care*, a census of all patients initiating ART in the 12 months of 2012 was taken (consistent with UNGASS/ PEPFAR). For *Viral load suppression at 6 months*, a census of all patients with available viral load results in ePMS from 1 October 2013 to 31 March 2014 was taken (with the additional criteria that the first viral load must have been conducted 3–12 months from ART start.)

### Calculation of national estimates

National estimates for adult and pediatric EWIs were calculated (Table 2) and are representative of Namibia’s public ART sites because data from all main and outreach sites in the country were abstracted.

**Table 2:** National Early Warning Indicator Summary Report.

Early Warning Indicator	EWI Target for all sites (time period)	Number of adult sites meeting EWI target (% of sites meeting target)	Number of pediatric sites meeting EWI target (% of sites meeting target)	National Adult Estimates % (CI or census result, as appropriate)	National Pediatric Estimates % (CI or census result, as appropriate)
**1. On-time pill pick-up**	Green: >90%	Green 41/193 (21%)	Green 54/162 (33%)	81.9% (81.1–82.8)*	82.4% (81.3–83.4)*
	Amber: 80–90%	Amber 57/193 (30%)	Amber 30/162 (18%)		
	Red: <80%	Red 93/193 (48%)	Red 77/162 (48%)		
	(1 Jan 2013 -)	Grey 2/193 (1%)	Grey 1/162 (<1%)		
**2. Retention in care**	Green: >85%	Green 65/193 (34%)	Green 60/162 (37%)	79% (11117/14095)	82% (846/1030)
	Amber: 75–85%	Amber 47/193 (24%)	Amber 9/162 (6%)		
	Red: <75%	Red 58/193 (30%)	Red 24/162 (15%)		
	(1 Jan 2012–31 Dec 2012)	Grey 23/193 (12%)	Grey 69/162 (43%)		
**3. Pharmacy stock-outs**	Green: 100%	Green 133/193 (69%)	Green 88/162 (54%)	94% (2168/2316)	88% (1720/1944)
	Red: <100%	Red 60/193 (31%)	Red 74/162 (46%)		
	(1 April 2013–31 Mar 2014)	Grey 0/193 (0%)	Grey 0/162 (0%)		
**4. Dispensing practices**	Green: 0%	Green 190/193 (98%)	Green 160/162 (99%)	0.01% (0.001–0.056)*	0.01% (0.001–0.069)*
	Red: >0%	Red 1/193 (<1%)	Red 1/162 (<1%)		
	(1 Jan 2013 -)	Grey 2/193 (1%)	Grey 1/162 (<1%)		
**5. Viral load suppression**	Green: >90%	Green 1/50 (2%)	Green 0/50 (0%)	-	-
	Amber: 75–90%	Amber 2/50 (4%)	Amber 3/50 (6%)		
	Red: <75%	Red 7/50 (14%)	Red 16/50 (32%)		
	(1 October 2012–31 Mar 2014)	Grey 40/50 (80%)	Grey 31/50 (62%)		
**5a. Viral load completion**	Green: ≥70%	Green 10/50 (20%)	Green 19/50 (38%)	42% (2303/5555)	66% (288/435)
	Red: <70%	Red 37/50 (74%)	Red 25/50 (50%)		
	1 October 2012–31 Mar 2014)	Grey 3/50 (6%)	Grey 6/50 (12%)		

CI—confidence interval

EWI—Early Warning Indicator

*National estimates were weighted by the number of active patients at each ART site

Point estimates for *On-time pill pick-up* and *Dispensing practices* were weighted by number of active patients at each ART site; confidence intervals for these EWIs were adjusted for clustering within ART sites. Weighting for *Retention in care*, *Pharmacy stock-out*, and *Viral load suppression* was not necessary because retention and viral load suppression are measured as a census of all appropriate data, and stock-outs are reports of actual drug shortages; confidence intervals for these EWIs are not reported because the data are measured by census.

### EWI trend analyses

Main and outreach site data for adults and pediatrics were compared for *On-time pill pick-up* and *Retention in care* using a Wilcoxon rank-sum test for independent samples. Data from main sites for adults and pediatrics were compared between 2012 and 2014 using the Wilcoxon signed-rank test in a matched analysis. All analyses were performed using STATA version 13 (College Station, TX: StataCorp LP).

## Results

**Fig 1:**
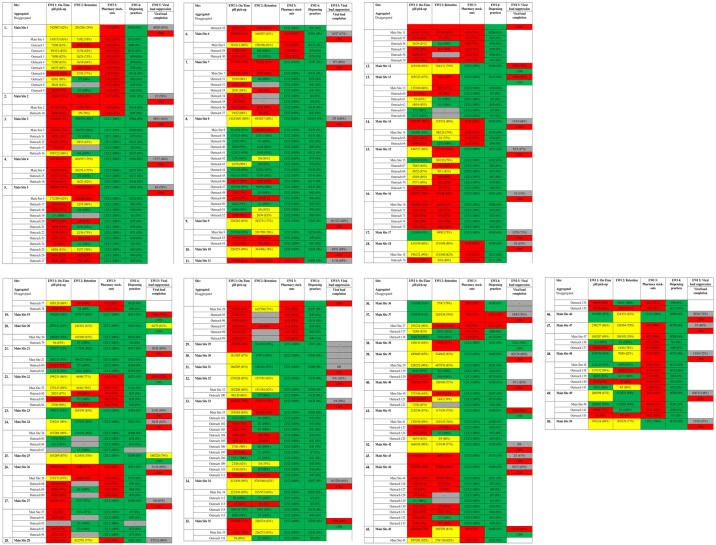
Adult Site-specific EWI Results. EWI—Early Warning Indicators Green indicates sites that achieved excellent performance, desired target level. Yellow indicates sites that achieved fair performance, progressing towards desired target level. Red indicates poor performance, below desired target. Gray indicates that data was not available from that site.

**Fig 2:**
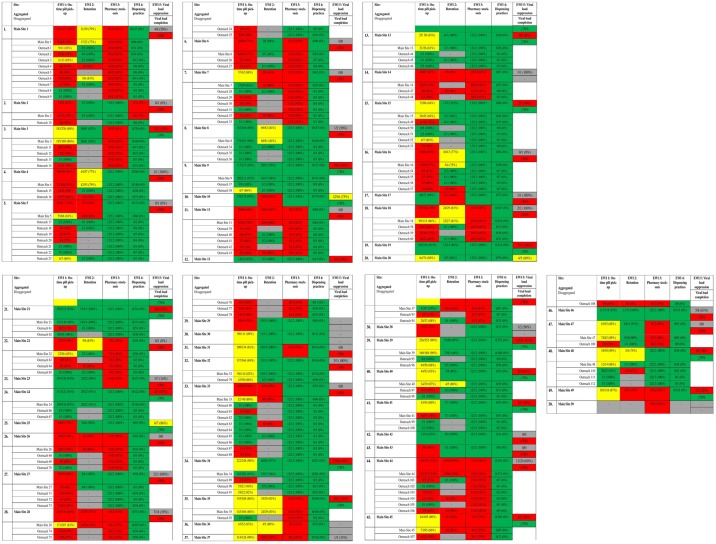
Pediatric Site-specific EWI Results. EWI—Early Warning Indicators Green indicates sites that achieved excellent performance, desired target level. Yellow indicates sites that achieved fair performance, progressing towards desired target level. Red indicates poor performance, below desired target. Gray indicates that data was not available from that site.

Namibia monitored five EWIs for both adult and pediatric patients in 2014: *On-time pill pick-up*, *Retention in care*, *Pharmacy stock-outs*, *Dispensing practices*, and *Viral load suppression at 6 months*. Data from 37,814 adult and 6,913 pediatric patient records were abstracted and analyzed from 50 main sites and 143 outreach sites. Site-specific EWI results are presented as both aggregated data (main site data that also included data for each main site’s respective outreach sites) and disaggregated data (main site and outreach site data that are presented separately) for adults in Fig 1 and for pediatrics in Fig 2. The national summary for the 2014 EWI monitoring round for both adults and pediatrics is presented in Table 2. Results for *Viral load suppression at 6 months* are presented with respective viral load completion rates for each site; only aggregated main site data are available for viral load suppression due to laboratory resource limitations at outreach sites.

### On-time pill pick-up

The national estimate for *On-time pill pick-up* was 81.9% (95% CI, 81.1%-82.8%) of patients picking up pills on time for adults and 82.4% (95% CI, 81.3%-83.4%) for pediatrics. For adults, 21% of sites achieved “excellent” performance (>90% of patients picking up pills on time) for *On-time pill pick-up*, 30% of sites had “fair” performance (80–90%), and 48% of sites had “poor” performance (<80%). For pediatrics, 33% of the sites achieved “excellent” performance, 18% of sites had “fair” performance, and 48% of sites had “poor” performance. (Table 2)

### Retention in care

The national estimate for *Retention in care* showed that 79% of adults were retained on ART after 12 months and 82% of pediatrics were retained on ART. For adults, 34% of the sites achieved “excellent” performance (>85% of patients retained after 12 months) for *Retention in care*, 24% of sites had “fair” performance (75–85%), and 30% of sites had “poor” performance (<75%). For pediatric sites, 37% of sites achieved “excellent” performance, 6% of sites had “fair” performance, and 15% of sites had “poor” performance. (Table 2)

### Pharmacy stock-outs

The national estimate for *Pharmacy stock-outs* is 94% of months without a stock-out for adults and 88% for pediatrics. For adults, 69% of sites achieved “excellent” performance with 100% of months without a pharmacy stock-out and 31% were classified as “poor” performance with <100% of months without a pharmacy stock-out. For pediatrics, 54% of sites achieved “excellent” performance and 46% of sites were classified as “poor” performance. (Table 2)

### Dispensing practices

The national estimates for *ARV dispensing practices* showed that 0.01% (95% CI, 0.001–0.056) of adults were dispensed mono- or dual-therapy and 0.01% (95% CI, 0.001–0.069) of pediatric patients were dispensed mono- or dual-therapy. For adults, 98% of the sites achieved “excellent” performance with 0% of patients dispensed mono- or dual therapy, and <1% were classified as “poor” performance with >0% of patients mono- or dual-therapy. For pediatric sites, 99% of achieved “excellent” performance and <1% were classified as “poor” performance. Only 1 of 18,165 adult patients and 1 of 5,447 pediatric patients were dispensed dual therapy; there was no dispensing of mono-therapy. (Table 2)

### Viral load suppression and completion at 6 months

For adults, only 20% achieved the target of ≥70% of viral loads completed for eligible patients, and 38% of pediatric sites achieved the target. The national estimate for viral load completion is 42% of eligible patients having viral loads completed for adults, and 66% for pediatrics. For *Viral load suppression* in adults, 2% of sites achieved “excellent” performance (>90% viral loads suppressed), 4% had “fair” performance (75–90% suppressed), 14% had “poor” performance (<75% suppressed), and 80% had insufficient data. For *Viral load suppression* in paediatrics, 0% of sites achieved “excellent” performance, 6% had “fair” performance, 32% had “poor” performance, and 62% had insufficient data. National level viral load suppression estimates were not calculated due to low levels of viral load completion.

### EWI trend analysis

A higher percent of patients picked up pills on time in main sites when compared to outreach sites for both adults (83% in main sites vs. 70% in outreach sites, p<0.001) and pediatrics (81% vs. 71%, p<0.001). There was no significant difference in *Retention in care* between main sites and outreach sites for adults (77% vs. 75%, p = 0.761) or pediatrics (83% vs. 82%, p = 0.214). (Table 3)

**Table 3:** Early Warning Indicator Trend Analysis Comparing Main and Outreach Site Performance.

Early Warning Indicator	Adult or Pediatric	Main Site Estimates % (CI)	Outreach Site Estimates % (CI)	p-value
**1. On-time pill pick-up**	Adults	83% (78–87)	70% (66–74)	<0.0001
	Pediatrics	81% (77–86)	71% (66–76)	<0.0001
**2. Retention in care**	Adults	77% (74–80)	75% (7–80)	0.7611
	Pediatrics	83% (78–88)	82% (72–92)	0.214
**3. Pharmacy stock-outs**	Adults	*Aggregate data only*	*Aggregate data only*	-
	Pediatrics	*Aggregate data only*	*Aggregate data only*	-
**4. Dispensing practices**	Adults	*Aggregate data only*	*Aggregate data only*	-
	Pediatrics	*Aggregate data only*	*Aggregate data only*	-
**5. Viral load suppression at 6 months**	Adults	*Aggregate data only*	*Aggregate data only*	-
	Pediatrics	*Aggregate data only*	*Aggregate data only*	-

CI—confidence interval

A comparison of main sites between 2012 and 2014 showed lower *On-time pill pick-up* rates in 2014 for both adults (87% in 2012 vs. 80% in 2014, p = 0.001) and pediatrics (87% vs. 80%, p<0.001). 2014 EWI also showed lower levels of *Retention in care* for adults (84% vs. 78%, p<0.001), but no difference for pediatrics (84% vs. 80%, p = 0.755). Both adult (99% vs. 94%, p = 0.002) and pediatric (97% vs. 90%, p = 0.0119) sites had fewer months without a stock-out in 2012 than in 2014. There were no significant differences in dispensing practices from 2012 to 2014 for either adults (p = 0.1573) or pediatrics (p = 0.1938). (Table 4)

**Table 4:** Early Warning Indicator Trend Analysis Comparing 2012 and 2014 Main Site Performances.

Early Warning Indicator	Adult or Pediatric	2012 Estimates % (CI)	2014 Estimates % (CI)	p-value
**1. On-time pill pick-up**	Adults	87% (85–90)	80% (76–84)	0.001
	Pediatrics	87% (85–90)	80% (77–84)	0.0009
**2. Retention in care**	Adults	84% (81–84)	78% (76–80)	<0.001
	Pediatrics	84% (79–88)	80% (74–87)	0.7553
**3. Pharmacy stock-outs**	Adults	99% (98–100)	94% (89–98)	0.0022
	Pediatrics	97% (94–99)	90% (85–96)	0.0119
**4. Dispensing practices**	Adults	<0.01%	<0.01%	0.1573
	Pediatrics	0.10%	0.10%	0.1938
**5. Viral load suppression at 6 months**	Adults	*2014 data only*	-	-
	Pediatrics	*2014 data only*	-	-

CI—confidence interval

## Discussion

This paper documents for the first time nationally representative EWI results from Namibia’s ART program. These data are significant in that to our knowledge no other country has published nationally representative EWI data to date. The analysis includes data for all 50 main public ART sites and all 143 outreach/IMAI sites. Data from 2014 were also compared with data from 2012 to assess trends in performance for the first time in Namibia.

*On-time pill pick-up* is an important measure of patient adherence that is associated with HIVDR [20–22], virological failure [23–26], and increased mortality [27–29]. Repeated treatment interruptions of 48 hours are strongly associated with virological failure and the emergence of HIVDR, therefore it is important to monitor timely pick-ups of ARVs in order to assure uninterrupted ARV adherence. [30] In Namibia, only 21% of adult sites and 33% of pediatric sites achieved the target of >90% of patients picking up pills on time. Similarly in Zimbabwe in 2013, only 22% of adult sites and 4% of pediatric sites achieved >90% of on-time pill pick-ups. [31] Also in Cameroon’s 2013 EWI, only 33% of sites achieved this target. [32] In the 2012 global report of EWI data in other African settings, only 15% of the 321 adult sites monitoring *On-time pill pick-up* achieved their target of >90%. [33] The 2016 global report of EWI data similarly showed that out of 5,027 clinics from 9 countries during 2010–2014, 85.5% (95% CI: 72.1–93.1%) of pill pick-ups were on time, falling short of the WHO target of 90%. [8] The Namibia national estimates of *On-time pill pick-up* for adults 81.9% (95% CI: 81.1–82.8) and pediatrics 82.4% (95% CI: 81.3–83.4) are similar to the overall estimates given in the global report, but are significantly higher than data reported from the African Region 69.9% (95% CI: 55.0–81.1%). [8] With these data, Namibia plans to design operational research to investigate site-level factors contributing to poor population adherence. Additionally, Namibia is conducting an operational research project with a short-messaging service (SMS) intervention to improve on-time pill pickup.

*Retention in care* 12 months after ART initiation is critical for preventing treatment interruptions [30, 34], as patients who disengage from care have increased risk for HIVDR and mortality. According to Brinkhof et al. [35], most mortality of LTFU patients occurs within the first six months of disengagement from care. In Namibia, only 34% of adult sites achieved the target of >85% retention at 12 months, with pediatric sites performing only slightly better. The percentage of sites achieving this target in Zimbabwe and Cameroon for adult patients in 2013 were 49% and 69%, respectively. [31–32] The 2016 global report of EWI data showed that out of 7,062 clinics in 50 countries during 2004–2014, 73.5% (95% CI: 66.5–79.6%) of individuals were retained in care 12 months after ART initiation, falling short of the WHO target of 85%. [8] The Namibia national retention rates for adults 79% and pediatrics 82% are higher than the overall estimates given in the global report, and are significantly higher than data reported from the African Region 67.6% (95% CI: 60.7–73.8%). [8] However, Namibia’s retention rates do not meet the WHO-recommended targets. It is possible that these low retention rates may be underestimates due to “silent transfers,” or patients who transfer to new clinics without informing their previous clinic. A 2013 tracing study in Malawi found that out of LTFU patients who were traced and alive, 56% were still taking ART from the original clinic or another clinic, and of those still engaged in care, only 24% reported any treatment gaps at all. [36] Namibia is engaged in a tracing study to determine what percentage of LTFU patients are truly disengaged from care. Namibia’s broad range of retention rates between ART sites suggest there may be factors at site-level that are influencing retention. Acting upon EWI data, Namibia has initiated defaulter tracing initiatives to examine reasons for and factors associated with LTFU.

*Pharmacy stock-outs* have a strong association with HIVDR emergence and poor viral load suppression, due to resulting treatment interruptions. In 2014, 69% of sites achieved 12 months with no pharmacy stock-outs for adults, and 54% of sites had no stock-outs for pediatrics. Namibia’s performance is relatively strong; in Zimbabwe’s 2013 EWI, 0% of adult sites achieved the target of no stock-outs, and Cameroon reported only 14% of sites with no stock-outs. [301–32] The 2016 global report of EWI data showed that out 1,703 clinics in 35 countries, 35.7% of clinics reported at least one stock out. Reasons for stock-outs in Namibia were poor inventory management practices, storage space constraints, and ARVs about to expire, which led to unusable ARVs at the sites. Namibia has been working on strengthening supervision by regional pharmacists in order to ensure proper drug forecasting, procurement, and supply distribution.

*Dispensing practices* are important to ensure that no mono- or dual-therapy is dispensed, which would lead to emergence of HIVDR. In 2014, very few adult or pediatric sites dispensed any mono- or dual-therapy in Namibia. These results indicate good training on national ART guidelines, and Namibia shows a strong performance among regional counterparts. Cameroon reported no dispensing of mono- or dual-therapy in any of its 2013 abstraction sites, while Zimbabwe reported that 86% of its adult sites and 82% of its pediatric sites achieved the target [31–32]. In other African countries, the reported percent of sites meeting the WHO target were 74% of 907 sites [33] and 85% of 13 sites [37]. The recent global EWI report also reported 99.1% (95% CI: 96–99.8%) of patients receiving appropriate regimens in 7,269 clinics from 52 countries (2005–2014). [8]

*Viral load suppression* is an important indicator for the prevention of HIVDR. However, viral load testing is still largely inaccessible and not routinely measured on a large scale in many African countries. [31–33] *Viral load suppression at 6 months* was able to be monitored for the first time in Namibia in 2014. Viral load completion rates were also calculated to evaluate the representativeness of the viral load suppression data. In the 2012 EWI data analysis phase, suppression rates were unreliable due to data entry issues at site-level. After a national training of data clerks, these data entry issues were believed to have been resolved. However, the 2014 EWI analysis showed low viral load suppression rates, with only 2% of adult sites achieving the target of >90% of viral loads suppressed, and 0% of pediatric sites achieving the target. These data do not accord with viral load suppression rates of 93% observed in HIVDR sentinel surveillance in Namibia. [38] It is likely that these rates are inaccurate because the respective viral load completion rates were very low (with only 20% of adult sites and 38% of pediatric sites achieving the WHO target of ≥70%). When the capability to perform viral load testing is limited, there may be preferential selection of sicker patients for conducting viral loads, thereby introducing a selection bias and artificially lowering overall suppression rates. Possible reasons for low viral load completion rates include: 1) ART sites not performing viral load testing on all eligible individuals, 2) laboratory results not being returned to the ART site, and 3) laboratory results not being entered into paper records or the electronic database. Based on this information, Namibia has begun to investigate reasons for low viral load completion rates at site-level in order to ensure that viral loads are properly performed on all eligible patients and entered into medical records.

Since this was the first year when the main site data were disaggregated from outreach site data, comparisons were made between main versus outreach sites to assess programmatic functioning at each level of care. Main sites performed significantly better than outreach sites in *On-time pill pickup*. There are at least three possibilities to explain this observation: 1) lack of data capture at outreach sites due to pill pickup data not being entered into the main site’s EDT, 2) delayed synchronization of information from EDT mobiles at outreach sites, or 3) actual missed pill pickups at outreach sites. Therefore, efforts should be made to investigate whether the observed worse pill pickup in outreach sites is due to data capture issues or due to limited access to care.

*Retention in care* rates between main and outreach sites were similar for both adults and pediatrics. This finding is somewhat surprising since decentralized sites might be expected to perform better at retaining patients in care due to convenience of location and smaller size of sites. For example, Chan et al. showed that patients treated at decentralized health centers in Malawi were 60% less likely to become lost to follow-up within the first 10 months of care. [39] A similar study in Kenya showed that pre-ART patients enrolled in a primary health facility had lower risk of becoming lost to follow-up than those enrolled at a secondary health facility. [40] However, more data are needed to identify the specific factors within decentralized sites that lead to optimal retention.

*Pharmacy stock-outs* represent aggregated data, where any stock-out at the main site would affect its respective outreach sites; therefore, no difference between main and outreach sites could be investigated. *Dispensing practices* remained excellent across both main and outreach sites, as well as across adult and pediatric sites, and no significant differences were found. *Viral load suppression* was available only for main sites, so no difference between main and outreach sites could be investigated.

Comparisons were made between 2014 and 2012 EWI data to assess national trends. *On-time pill pick-up*, *Retention in care*, and *Pharmacy stock-outs* all appeared to worsen from 2012 to 2014 in adult sites; and *On-time pill pick-up* and *Pharmacy stock-outs* appeared to worsen in pediatric sites. This observation may be due weakening of ART records. However, as ART is scaled up over time, it is possible that the health system is not able to keep up with the growing demands for care. A similar trend was observed in Cameroon, which saw worsening performance between 2008 and 2010 in four of five EWIs measured; authors cite insufficient resources and trained personnel to support the rapid scaling up of ART services as a potential reason for this decline. [41]

One important limitation was ART data quality. Data validation between national databases was labor intensive due to difficulty linking EDT and ePMS records, which oftentimes have incongruent information. As decentralization of ART services continues, data quality should be strengthened at IMAI and outreach sites, since many outreach sites had missing data. In the future, Namibia plans to bolster capacity-building for adequate data entry, capture, and abstraction by increasing pharmacy staff and data clerks.

However, despite these limitations, this year’s EWI round was undoubtedly the most inclusive and exhaustive EWI analysis in Namibia to date. The national program has successfully responded to the recommendations provided by the previous rounds of EWIs in Namibia, making the collection of novel and important data possible. Viral load suppression rates, which are commonly unavailable on such a large scale in other African countries due to resource limitations, were included for the first time in the 2014 EWI analysis. Additionally, data for outreach sites were successfully abstracted and compared to main site data for the first time in Namibia.

The successful 2014 EWI exercise, built upon three previous rounds of EWIs [13–14], provides Namibia a solid evidence base, which can be used to make national statements about programmatic functionality in the context of HIVDR. Significantly, EWI trend analyses—analyzing EWI data over time—can be performed in Namibia moving forward, as adult and pediatric data from all 50 main ART sites are now regularly included in the national exercise. This evidence base will contextualize results from Namibia’s surveys of HIVDR, which involve HIV genotype testing. Namibia has begun to analyze the private sector data to investigate prescribing practices and on-time pill pickup.

The EWI abstraction process has mobilized the national ART program and its partners to institute minor adjustments in existing databases, which will continue to facilitate abstraction of WHO recommended EWIs in the future. Importantly, these four successful rounds of EWI monitoring have highlighted the potential for HIVDR emergence in Namibia. In response to these data, the national program has engaged sites in dialogue regarding the need to strengthen adherence and retention of patients on ART and has implemented numerous operational research projects. Additionally, Namibia plans to investigate sites with sub-optimal results as well as sites functioning well in order to learn lessons and replicate in other sites. The EWI collection process and results will optimize patient care and will support the HIV prevention and care program in Namibia in making evidence-based recommendations and taking actions to minimize the emergence of preventable HIVDR.

EWIs in Namibia have been integrated into the routine Monitoring and Evaluation activities of the MoHSS HIV care and treatment program, thereby ensuring sustainability into the future. EWIs are routinely monitored at site level and reported as an ongoing activity with continuous validation along with annual national reporting.
